# Molecular Mechanisms of West Nile Virus Pathogenesis in Brain Cells

**DOI:** 10.3201/eid1104.041076

**Published:** 2005-04

**Authors:** Wee-Lee Koh, Mah-Lee Ng

**Affiliations:** * National University of Singapore, Singapore

**Keywords:** Flaviviruses, gene expression, microarray, real-time PCR, human glioblastoma cells, neuronal damage, dispatch

## Abstract

We analyzed the response of human glioma cells to West Nile virus infection by investigating host transcriptional changes. Changes in expression of 23 genes showed similarities to those in other neurodegenerative diseases. These changes may be useful as potential biomarkers and elucidate novel mechanisms behind the neuropathology of infection with this virus.

West Nile virus (WNV), a member of the family *Flaviviridae*, is the etiologic agent of West Nile fever. Since WNV is neurotropic, severe human meningoencephalitis is a common complication of infection and results in a considerable number of deaths. The medulla of the brainstem in the central nervous system (CNS) is the primary target of WNV (*1*).

WNV replicates in a wide variety of cell types, and studies have traditionally been carried out in Vero (green monkey kidney) and C6 (mosquito) cells. However, little work has been done with CNS cells. We conducted a global transcriptional analysis of human glioblastoma cell response to infection with WNV during peak virus production to determine the crucial virus-host interactions that take place during a severe neuroinvasive attack and identify putative mechanisms involved in WNV pathogenesis. The factors governing the development of neurologic disease, host immune response, patterns of clinical features, and outcomes are poorly understood in those infected with neurotropic flaviviruses (*2*).

A total of 173 genes were differentially expressed, many of which were not found in previous transcriptional studies of other flaviviruses (*3*). From these, 23 genes were identified that may play a role in cellular neurodegeneration. These novel changes induced by WNV may serve as biomarkers and help explain the neuropathologic features observed.

## The Study

Most laboratory studies of WNV infections have been carried out in animal cell lines or human cell lines of non-CNS origins. In this study, human glioblastoma (A172) cells were found to be a useful laboratory model for investigating WNV infections. A172 (human glioblastoma) cells were maintained at 37°C in Dulbecco modified Eagle medium (Sigma, St. Louis, MO, USA) supplemented with 10% fetal calf serum. Confluent monolayers of A172 cells were infected with the Sarafend strain of WNV at a multiplicity of infection of 1. Twenty-four hours after infection, cells showed signs of cytopathic effects (cell-rounding) and produced high virus titer (10^8^ PFU/mL). This demonstrated the highly susceptible nature of the neuroglial cells to WNV infection. Batches of cells were infected for microarray experiments, and a quantitative polymerase chain reaction was used to verify the reproducibility of the changes in gene expression.

Total cellular RNA was extracted from mock-infected and infected cells by using the RNeasy Mini kit and QIAshredder (Qiagen, Hilden, Germany). The CyScribe first-strand cDNA labeling kit (Amersham Biosciences, Piscataway, NJ, USA) was used to incorporate fluorescent Cy3-dCTP or Cy5-dCTP (Amersham Biosciences) into cDNA probes. The probes were subsequently purified by using CyScribe GFX purification columns (Amersham Biosciences). Equal amounts of labeled cDNA probes (≈25 pmol) were combined for microarray hybridizations. Human 1A microarrays (Agilent Technologies, Palo Alto, CA, USA) were used, and hybridizations were performed on a Lucidea SlidePro Hybridizer (Amersham Biosciences). The microarray experiment was carried out in triplicate: 1 of the microarrays was with a dye-swap labeling to prevent skew in the results due to bias in CyDye incorporation.

Analyses of the scanned microarray images were performed with BRB ArrayTools version 3.1 (developed by R. Simon and A.P. Lam, National Cancer Institute, Bethesda, MD, USA, and available at http://linus.nci.nih.gov/BRB-ArrayTools.html), and normalized by using the Lowess method. A stringent lower limit threshold was set at 3 standard deviations of the pixel intensities of the negative control spots, and images were screened for changes in expression values of at least 2-fold. The differentially regulated genes were separately uploaded into EASE (*4*) to determine the biologic themes that were significantly overrepresented (Fisher exact test with p values < 0.01). A total of 173 cellular genes were identified by ArrayTools to be differentially expressed in the WNV-infected A172 cells. EASE clustered 39 of the upregulated genes and 41 of the downregulated genes into specific functional groups (available at http://sps.nus.edu.sg/~kohweele/awn_genes.htm).

Functional classes that were found to be enriched in the upregulated genes encompassed those related to immunity, responses to external stimulus and pathogens, and apoptosis. Genes relating to the ubiquitin cycle, transcription regulation, and other physiologic processes were also identified by EASE. Functional classes that were downregulated were not commonly observed in a virus infection system. For instance, genes relating to the mitochondria, ribosomes, and protein biosynthesis were highly overrepresented in down regulation (available at http://sps.nus.edu.sg/~kohweele/awn_genes.htm). From this set of genes, a group of 23 genes that may provide the molecular basis for the observed pathogenesis in the A172 cells was identified (Table 1).

**Table 1:** Differentially regulated genes involved in pathogenesis of A172 cells infected with West Nile virus

Gene	Gene name	Fold change
Immune response related		
OAS3	2'-5'-oligoadenylate synthetase 3, 100 kDa	2.32
OASL	2'-5'-oligoadenylate synthetase-like	3.46
FIT1	Interferon-induced protein with tetratricopeptide repeats 1	10.74
IFIT2	Interferon-induced protein with tetratricopeptide repeats 2	3.76
IFI27	Interferon, α-inducible protein 27	4.03
IFITM1	Interferon-induced transmembrane protein 1 (9-27)	12.00
IFITM2	Interferon-induced transmembrane protein 2 (1-8D)	3.04
G1P2	Interferon, α-inducible protein (clone IFI-15K)	9.50
HLA-C	Major histocompatibility complex, class I, C	2.20
INDO	Indoleamine-pyrrole 2,3 dioxygenase	3.38
PTX3	Pentaxin-related gene, rapidly induced by interleukin-1β	3.44
Apoptosis related		
TNFSF14	Tumor necrosis factor (TNF) (ligand) superfamily, member 14	2.19
NFKBIA	Nuclear factor of kappa light polypeptide gene enhancer	4.13
TRAF1	TNF receptor-associated factor 1	2.01
SAT	Spermidine/spermine N1-acetyltransferase	2.18
Mitochondria related		
SDHC	Succinate dehydrogenase complex, subunit C	–2.31
COX5B	Cytochrome c oxidase subunit Vb	–2.13
COX6B	Cytochrome c oxidase subunit VIb	–2.41
ATP5G1	ATP synthase, mitochondrial F0 complex, subunit c, isoform 1	–2.64
ATP5C1	ATP synthase, mitochondrial F1 complex, γ polypeptide 1	–3.82
ATP5J	ATP synthase, mitochondrial F0 complex, subunit F6	–2.11
ATP5B	ATP synthase, mitochondrial F1 complex, β polypeptide	–2.17
ATP5A1	ATP synthase, mitochondrial F1 complex, α subunit, isoform 1	–2.21
ATP5O	ATP synthase, mitochondrial F1 complex, O subunit	–2.00
ATP5F1	ATP synthase, mitochondrial F0 complex, subunit b, isoform 1	–2.43
PRDX5	Peroxiredoxin 5	–2.74
PRDX3	Peroxiredoxin 3	–2.27
Protein biosynthesis related		
NACA	Nascent-polypeptide-associated complex polypeptide	–2.17

A quantitative reverse transcription–polymerase chain reaction (qRT-PCR) was carried out to ensure an independent assessment of the microarray results. Genes for the qRT-PCR were selected to represent the broad spectrum of identified functional classes from the microarrays. The hypoxanthine guanine phosphoribosyltransferase gene was used as an internal control (primers for the PCR can be found at http://sps.nus.edu.sg/~kohweele/awn_genes.htm). RNA was reverse-transcribed by using SuperScript III (Invitrogen, Carlsbad, CA, USA), and a real-time PCR was carried out with Platinum SYBR Green (Invitrogen). A negative template control that contained all SYBR green reagents except DNA was performed in parallel on the iCycler iQ (Bio-Rad Laboratories, Hercules, CA, USA). The results corroborated the microarray data, thereby verifying the accuracy of the statistical analysis (Table 2). However, the qRT-PCR showed greater dynamism in fold changes than the microarray results because of the greater sensitivity of PCR compared with fluorescent detection.

**Table 2:** Comparison of gene expression changes between microarray and qRT-PCR in A172 cells infected with West Nile virus*

Gene	Gene name	Microarray fold change	RT-PCR fold change
ARHI	DIRAS family, GTP-binding RAS-like 3	–2.72	–2.55
ATP5J	ATP synthase, mitochondrial F0 complex, subunit F6	–2.11	–2.60
CEB1	Hect domain and RLD 5	2.32	42.22
DNAJB1	DnaJ (Hsp40) homolog, subfamily B, member 1	–1.97	–2.14
DUSP1	Dual specificity phosphatase 1	1.92	5.66
EGR1	Early growth response 1	4.79	8.57
EIF4G2	Eukaryotic translation initiation factor 4 γ, 2	–2.11	–7.77
FLJ13855	Hypothetical protein FLJ13855	2.05	3.85
FOSL1	FOS-like antigen 1	2.08	6.50
IFITM1	Interferon-induced transmembrane protein 1 (9-27)	12.03	527.61
LTA4H	Leukotriene A4 hydrolase	–2.02	–8.10
RPL5	Ribosomal protein L5	–2.97	–9.03
RPL7A	Ribosomal protein L7a	–2.03	–3.42
RPLP0	Ribosomal protein, large, P0	–2.15	–1.52
TFPI2	Tissue factor pathway inhibitor 2	5.21	11.58

*qRT-PCR, quantitative reverse transcription–polymerase chain reaction.

## Conclusions

In this study, WNV infection of human brain glioma cells showed advanced cytopathic effects within 24 h after infection and produced high virus yields. This demonstrated that human glioma cells from CNS are susceptible to WNV infection and are suitable for the study of viral pathogenesis.

The activation of the innate antiviral immune response pathways is often the primary cause of pathologic effects. The presence of double-stranded RNA replication complexes from viral origins causes the transcriptional activation of the interferon-α/β (IFN-α/β) or type-I IFN pathways (*5*). In this study on glioma cells, the activation of numerous interferon-induced proteins (such as IFIT1, IFIT2, IFI27, IFITM1, IFITM2, and G1P2) lends support to this mechanism of pathogenicity. Glial cells are useful in this study because they are immune cells of CNS origin. Activated glial cells have macrophagic activity and are primed to respond to the virus, therefore allowing the display of immune-mediated neuropathologic changes that reflect conditions in the natural CNS host cells. Glial cells can also activate the type-II (IFN-γ) pathway and modulate the immune response by regulating cell trafficking of various leukocytes, including macrophage activation and stimulation of specific T cells responsible for cytotoxic immunity (*6*).

An example of this activation was finding that the HLA-C gene coding for the major histocompatibility complex class I (MHC-I) antigens was upregulated in the A172 cells. Peptides derived from endogenous intracellular proteins are generally bound by the MHC-I molecules for presentation, thus paving the way for cell cytotoxicity in cellular immunity. In mice, the targeted killing of WNV-infected cells by CD8+ T cells may result in the severe neurologic disease often observed in WNV infections (*7*).

In addition, indoleamine 2,3 dioxygenase (INDO) was observed to be upregulated in WNV-infected A172 cells. Increased production of INDO by glial cells causes neuronal injury in neuroinflammatory diseases (*8*). The upregulation of the pentaxin-related gene (PTX3) is also implicated in local tissue damage through the amplification of inflammation in innate immunity (*9*).

A group of genes causing apoptosis was also found to be upregulated, thus elucidating pathways linking virus replication to apoptosis. These genes include the tumor necrosis factor superfamily (TNFSF14), nuclear factor of κ light-chain gene (NFKBIA), TNF receptor-associated factor (TRAF1), and spermidine/spermine N1-acetyltransferase (SAT). This highly conserved process of cellular self-destruction serves to limit the spread of WNV (*10*).

A major group of genes relating to mitochondria was found to be downregulated. Mitochondrial defects due to respiratory-chain dysfunction and free-radical formation have been associated with neurodegenerative diseases such as Huntington disease, Parkinson disease, and Friedreich ataxia (*11*). Neurologic symptoms of these diseases were also observed in WNV-infected patients (*12*), suggesting similar neurodegenerative pathways.

The activity of genes belonging to the energy synthesis pathways was decreased. These genes included succinate dehydrogenase (SDHC), cytochrome c oxidase (COX5B/COX6B), and various genes of the ATP synthase complex (ATP5G1, ATP5C1, ATP5J, ATP5B, ATP5A1, ATP5O, and ATP5F1). Decreased energy production from the downregulation of these genes is known to cause severe neurodegeneration (*13*). Two antioxidant enzymes of the peroxiredoxin family (PRDX5 and PRDX3) were also downregulated. The increase in oxidative stress induced by reactive oxygen species can create a proinflammatory condition that results in CNS pathology and leads to Alzheimer disease and Down syndrome (*14*). Downregulation of the nascent polypeptide-associated complex (NACA) can also lead to similar neurodegeneration (*15*).

In summary, this global transcriptional study showed a complex network of WNV-induced A172 cell interactions during infection. The examination of glial A172 cell response has provided insights into the molecular mechanisms behind the observed neuronal pathology in WNV encephalitis.

## References

[1] Sampson BA, Ambrosi C, Charlot A, Reiber K, Veress JF. The pathology of human West Nile virus infection. Hum Pathol. 2000;31:527–31. 10.1053/hp.2000.8047 10836291

[2] Solomon T, Winter PM. Neurovirulence and host factors in flavivirus encephalitis—evidence from clinical epidemiology. Arch Virol Suppl. 2004;18:61–70. 10.1007/978-3-7091-0572-6_14 15119771

[3] Warke RV, Xhaja K, Martin KJ, Fournier MF, Shaw SK, Brizuela N, Dengue virus induces novel changes in gene expression of human umbilical vein endothelial cells. J Virol. 2003;77:11822–32. 10.1128/JVI.77.21.11822-11832.2003 14557666 PMC229255

[4] Hosack DA, Dennis G, Sherman BT, Lane H, Lempicki RA. Identifying biological themes within lists of genes with EASE. Genome Biol. 2003;4:R70. 10.1186/gb-2003-4-10-r70 14519205 PMC328459

[5] Samuel CE. Antiviral actions of interferons. Clin Microbiol Rev. 2001;14:778–809. 10.1128/CMR.14.4.778-809.2001 11585785 PMC89003

[6] Salmaggi A, Gelati M, Dufour A, Corsini E, Pagano S, Baccalini R, Expression and modulation of IFN-gamma-inducible chemokines (IP-10, Mig, and I-TAC) in human brain endothelium and astrocytes: possible relevance for the immune invasion of the central nervous system and the pathogenesis of multiple sclerosis. J Interferon Cytokine Res. 2002;22:631–40. 10.1089/10799900260100114 12162873

[7] Shrestha B, Diamond MS. Role of CD8+ T cells in control of West Nile virus infection. J Virol. 2004;78:8312–21. 10.1128/JVI.78.15.8312-8321.2004 15254203 PMC446114

[8] Grant R, Kapoor V. Inhibition of indoleamine 2,3-dioxygenase activity in IFN-gamma stimulated astroglioma cells decreases intracellular NAD levels. Biochem Pharmacol. 2003;66:1033–6. 10.1016/S0006-2952(03)00464-7 12963490

[9] Bottazzi B, Vouret-Craviari V, Bastone A, de Gioia L, Matteucci C, Peri G, Multimer formation and ligand recognition by the long pentraxin PTX3: similarities and differences with the short pentraxins C-reactive protein and serum amyloid P component. J Biol Chem. 1997;272:32817–23. 10.1074/jbc.272.52.32817 9407058

[10] Chu JJH, Ng ML. The mechanism of cell death during West Nile virus infection is dependent on initial infectious dose. J Gen Virol. 2003;84:3305–14. 10.1099/vir.0.19447-0 14645911

[11] Schapira AH. Mitochondrial dysfunction in neurodegenerative disorders. Biochim Biophys Acta. 1998;1366:225–33. 10.1016/S0005-2728(98)00115-7 9714816

[12] Burton JM, Kern RZ, Halliday W, Mikulis D, Brunton J, Fearon M, Neurological manifestations of West Nile virus infection. Can J Neurol Sci. 2004;31:185–93. 15198442 10.1017/s0317167100053828

[13] Eng C, Kiuru M, Fernandez MJ, Aaltonen LA. A role for mitochondrial enzymes in inherited neoplasia and beyond. Nat Rev Cancer. 2003;3:193–202. 10.1038/nrc1013 12612654

[14] Kim SH, Fountoulakis M, Cairns N, Lubec G. Protein levels of human peroxiredoxin subtypes in brains of patients with Alzheimer's disease and Down syndrome. J Neural Transm Suppl. 2001;61:223–35. 11771746 10.1007/978-3-7091-6262-0_18

[15] Kim SH, Shim KS, Lubec G. Human brain nascent polypeptide-associated complex alpha subunit is decreased in patients with Alzheimer's disease and Down syndrome. J Investig Med. 2002;50:293–301. 10.2310/6650.2002.33287 12109594

